# Audiovisual Integration During Joint Action: No Effects for Motion Discrimination and Temporal Order Judgment Tasks

**DOI:** 10.3389/fpsyg.2020.00079

**Published:** 2020-02-04

**Authors:** Basil Wahn, Jill A. Dosso, Alan Kingstone

**Affiliations:** Department of Psychology, University of British Columbia, Vancouver, BC, Canada

**Keywords:** multisensory integration, social cognition, joint action, motion, temporal, task co-performance

## Abstract

In daily life, humans constantly process information from multiple sensory modalities (e.g., visual and auditory). Information across sensory modalities may (or may not) be combined to form the perception of a single event via the process of multisensory integration. Recent research has suggested that performing a spatial crossmodal congruency task jointly with a partner affects multisensory integration. To date, it has not been investigated whether multisensory integration in other crossmodal tasks is also affected by performing a task jointly. To address this point, we investigated whether joint task performance also affects perceptual judgments in a crossmodal motion discrimination task and a temporal order judgment task. In both tasks, pairs of participants were presented with auditory and visual stimuli that might or might not be perceived as belonging to a single event. Each participant in a pair was required to respond to stimuli from one sensory modality only (e.g., visual stimuli only). Participants performed both individual and joint conditions. Replicating earlier multisensory integration effects, we found that participants' perceptual judgments were significantly affected by stimuli in the other modality for both tasks. However, we did not find that performing a task jointly modulated these crossmodal effects. Taking this together with earlier findings, we suggest that joint task performance affects crossmodal results in a manner dependent on how these effects are quantified (i.e., via responses time or accuracy) and the specific task demands (i.e., whether tasks require processing stimuli in terms of location, motion, or timing).

## 1. Introduction

Humans constantly process information from several sensory modalities (e.g., touch, vision, audition). This information may (or may not) be combined to form a unitary percept via the process of multisensory integration. Previous research has investigated several factors that could affect this integration process ranging from where and when the stimuli occur (Meredith et al., 1987; Meredith and Stein, 1996; Guski and Troje, 2003; Holmes and Spence, 2005) to the attentional demands placed on the observer (Bertelson et al., 2000; Vroomen et al., 2001; Alsius et al., 2005; Santangelo and Spence, 2008, 2009; Santangelo and Macaluso, 2012; Vercillo and Gori, 2015; Wahn and König, 2015a,b, 2016; Wahn et al., 2017b).

To date, however, only a handful of studies have investigated whether social factors (e.g., performing a task with another person) affect multisensory integration (Heed et al., 2010; Wahn et al., 2017a). This comes something as a surprise given that past work on joint action has demonstrated that social factors can have a significant impact on how individuals perceive an isolated visual event (Sebanz et al., 2003, 2006; Knoblich and Sebanz, 2006; Böckler et al., 2012; Karlinsky et al., 2017; Vesper et al., 2017). Also, people in everyday life routinely perform multisensory tasks with, or in the presence of, others. For instance, when eating a meal with a friend, visual, tactile, smell, and taste information are combined; and when attending a concert both visual (e.g., seeing the musicians) and auditory information (e.g., hearing the music) are processed in the presence of others.

To the best of our knowledge, the first study (Heed et al., 2010) that investigated the relationship between multisensory integration and joint task performance involved a tactile spatial localization task. Heed et al. (2010) required participants to indicate the location of a tactile stimulus while a visual stimulus was presented simultaneously in either the same (congruent) or different (incongruent) location. Past work has demonstrated that when participants perform this type of task alone, responses to the tactile stimulus are slower and less accurate if the visual stimulus appears at an incongruent location (Spence et al., 2004). Heed et al. (2010) found that this congruency effect was reduced when performing the task jointly, as the participant performing the tactile task “off-loaded” attending to the visual distractor to their partner. As a potential mechanism to explain this effect, Heed et al. (2010) suggested that the participant performing the tactile task co-represented (Sebanz et al., 2003) the partner's task and could hence better filter out the visual distractors from their own task representation. The process of co-representation (i.e., that co-actors take into account each other's tasks) has been proposed to occur automatically whenever co-actors perform tasks jointly (Sebanz et al., 2003) and to form the basis for more complex joint actions (Vesper et al., 2010). In a recent study, we replicated the finding by Heed et al. (2010) in a joint audiovisual congruency task (Wahn et al., 2017a). That is, we found that the negative effect of an incongruent visual stimulus on sound localization was reduced for participants performing the task jointly. Relatedly, Sellaro et al. (2018) found that such a division of labor of tasks also reduced interference in a purely visual picture–word interference task (for a recent review about the benefits of labor division in joint tasks, see Wahn et al., 2018).

While the above research has demonstrated that performing a task jointly does affect audiovisual and visuotactile integration in a spatial congruency task (Heed et al., 2010; Wahn et al., 2017a), it has not been investigated whether the effect of joint performance on multisensory integration can be generalized to other situations, particularly whether the results extend beyond the presentation of two solitary, static stimulus events. The stimuli one routinely encounters in everyday life are normally in motion because we are often in motion (e.g., walking, moving our head, and shifting our gaze several times a second) and the world around us is in motion, too (e.g., living animals move, water flows, and trees sway in the wind). An important extension of the previous work then is to test if multisensory integration with moving stimuli is affected by joint performance. That is, investigating this question would be informative as to whether multisensory integration of *moving* stimuli is also affected by joint task performance or whether the effect of joint task performance is specific to stationary spatial stimuli.

Soto-Faraco et al. (2002) introduced an audiovisual motion congruency task that is conceptually very similar but qualitatively distinct from the crossmodal congruency task with static stimuli (Soto-Faraco et al., 2002, 2004), but to date it has only been tested with individual, isolated participants. In the typical audiovisual motion task, a participant receives visual and auditory stimuli that either move together in the same direction (congruent presentations) or in opposite directions (incongruent presentations). The critical task is to judge the movement direction of the auditory stimuli. Results indicated that participants often failed to correctly identify the direction of sound motion when the direction of the visual motion was incongruent (e.g., leftward auditory motion and rightward visual motion). We viewed this paradigm as a logical next step to test whether joint task performance affects audiovisual integration using a task involving more ecologically valid stimuli (i.e., motion stimuli). That is, the audiovisual motion congruency task used by Soto-Faraco et al. (2002) represented only a minimal change (i.e., static stimuli are replaced by moving stimuli) relative to our earlier study using a spatial audiovisual congruency task (Wahn et al., 2017a).

Importantly, past work has also demonstrated that the effect of multisensory integration varies with the nature of the task. For instance, as discussed above, when judging the spatial direction of two auditory stimuli, irrelevant and incongruent visual stimuli have a negative effect on performance. Note, when the task is reversed, and one is required to determine the direction of two visual stimuli, incongruent auditory motion has no impact on performance. In general, the explanation for this asymmetry is that multisensory integration is preferentially biased toward the modality that provides the most reliable signal for the task at hand, in this case, spatial direction. In other words, vision provides a more reliable spatial signal than does audition, a point we are all too familiar with when we are trying to determine in a group whose phone is ringing; it is only when we see a person move that we localize the sound. Critically, this advantage of a visual signal over an auditory signal reverses when the task is to judge when, rather than where, two events have occurred. This was demonstrated by Morein-Zamir et al. (2003) who asked participants to judge which of two visual stimuli appeared first on a computer screen. They found that performance improved when an auditory click trailed the second visual stimulus, as if the second visual event was pulled toward the trailing auditory click. As the paradigm used by Soto-Faraco et al. (2002) can be readily adapted to that of Morein-Zamir et al. (2003), we examined if a joint task manipulation affects both dynamic spatial judgments and temporal judgments. That is, adapting the paradigm used by Soto-Faraco et al. (2002) to that of Morein-Zamir et al. (2003) only involves minimal changes (i.e., instead of judging the direction of two rapidly presented stimuli, participants are required to judge the temporal order of two stimuli), allowing for the specific targeting of the question of whether temporal judgments are affected by joint task performance in a within-subject design. In doing so, we can also assess whether potential effects of joint task performance are comparable both in situations where vision affects auditory judgments (i.e., sound direction) and when audition affects visual judgments (i.e., visual timing). Finally, it is worth noting that there is currently uncertainty in the literature as to whether joint task performance on multisensory integration affects how quickly people respond, their accuracy of response, or both. For instance, Heed et al. (2010) used inverse efficiency scores (i.e., a combined measure of response times and accuracy) to analyze their data, rendering the speed/accuracy question equivocal. Wahn et al. (2017a) analyzed response times and accuracy separately and found that joint task performance only affected response times. As crossmodal congruency effects on response times are often vulnerable to alternative explanations that do not demand an explanation in terms of multisensory integration (e.g., a race model explains why two congruent signals may result in faster responses time than either of them alone, see Miller, 1982; Stevenson et al., 2014) we aimed to test whether performing a task jointly affects perceptual judgments (i.e., perceptual accuracy) rather than response times. Crucially, in both tasks mentioned above (i.e., the motion discrimination task and temporal order judgment task), crossmodal effects were quantified via response accuracies.

To summarize, the current work aims to extend previous research on multisensory integration and joint task performance (Heed et al., 2010; Wahn et al., 2017a) in three ways: (1) through the use of moving rather than static stimuli in a crossmodal congruency task, (2) by investigating temporal crossmodal effects, and (3) by using tasks that quantify crossmodal effects with regard to response accuracy (rather than response time).

## 2. Methods

### 2.1. Participants

Nineteen pairs of students (32 female and 6 male, *M* = 19.58 years, *SD* = 1.44 years) of the University of British Columbia participated in the present study. The participants provided their written informed consent to participate in this study and received course credits as compensation for their participation.

### 2.2. Experimental Setup

**Figure 1:**
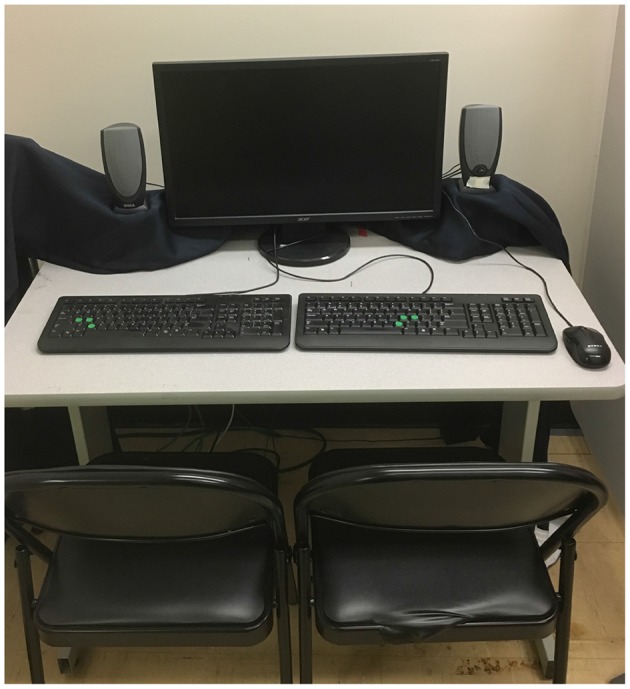
Experimental setup. Response keys were marked by green stickers on the keyboards.

Pairs of participants were seated next to each other, 60 cm from a computer screen (resolution: 1920 x 1000 pixels, 64.13 x 33.40 visual degrees, 60 Hz refresh rate, model: ACER V243H, 24 inches) so that when they looked straight ahead they could see the left or right edge of the computer screen, respectively. The auditory stimuli were received via speakers (model: Dell A215) placed next to the computer screen. The speakers were positioned at a height so that the middle of the speakers would align with the middle of the screen and were about 80 cm apart from each other. In front of each participant, a QWERTY keyboard was positioned for making responses (see Figure 1 for an overview of the experimental setup). The experiment was run on an Apple Mac Mini (2012 model), and we used its internal sound card to play the auditory stimuli.

### 2.3. Experimental Procedure

Each pair of participants performed the motion discrimination task and the temporal order judgment task. The order of tasks was counterbalanced across pairs so that half of the pairs started with the motion discrimination task and the remaining half began with the temporal order judgment task. In the following section we describe the procedure for each task separately. As a point of note, the participant performing the auditory motion discrimination task also performed the visual temporal order judgment task. The reasoning for this design choice was that, in both tasks, the crossmodal effects were expected to occur, and we planned to correlate the size of the crossmodal effects and the size of social effects across tasks.

#### 2.3.1. Motion Discrimination Task

**Figure 2:**
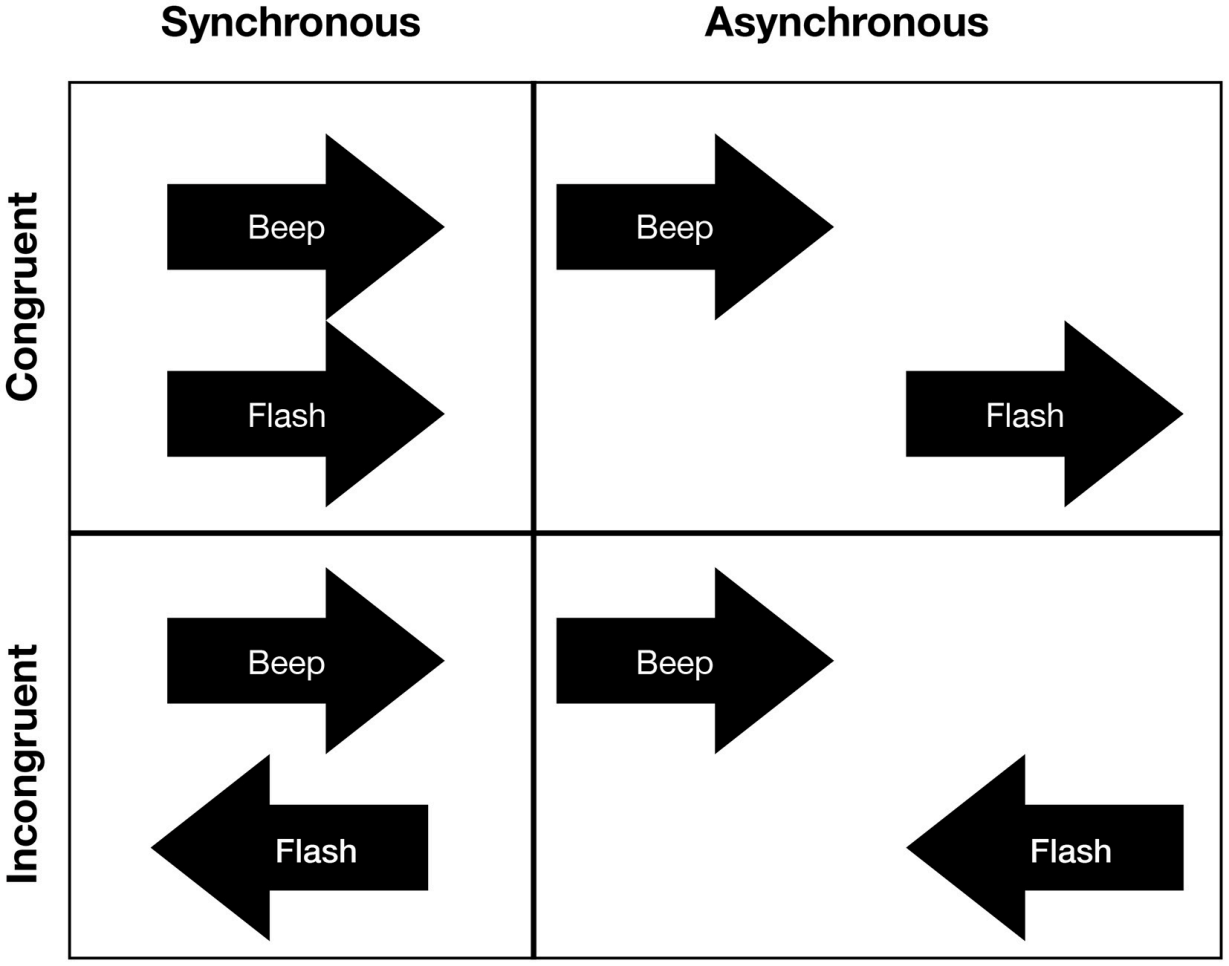
Stimuli combinations for the motion discrimination task: Auditory and visual stimuli could either synchronously (upper left) or asynchronously (upper right) move in the same direction or opposite directions (lower left and lower right). If stimuli were presented asynchronously, the first flash was presented 300 ms after the second auditory stimulus.

In the motion discrimination task, two beeps (duration: 50 ms) were presented, one from each speaker, one after the other in a rapid sequence (interstimulus interval: 100 ms) to create the apparent perception that stimuli were moving either in the left or right direction. The frequency of the two beeps was randomly selected out of a set of three frequencies (450, 500, and 550 Hz). Simultaneously with the presentation of the beeps, two flashes (duration: 50 ms) were presented that moved either in the same direction (congruent presentation) or opposite directions (incongruent presentation) of the auditory stream. The flashes (radius: 1.34 visual degrees) were presented at a distance of 15 visual degrees from a fixation dot (radius: 0.53 visual degrees) that was positioned in the center of the computer screen. In control trials, the flashes and beeps were presented asynchronously (for an overview of stimuli combinations see Figure 2). That is, in the asynchronous trials, the presentation of the flashes began 300 ms after the second beep was presented. For each trial, we randomly selected whether stimuli were presented synchronously or asynchronously.

One of the participants in the pair was required to perform the same auditory motion discrimination task as in the original study by Soto-Faraco et al. (2002). That is, they were required to indicate the movement direction of the beeps. For participants that sat on the left, they indicated the motion direction using the “A” key (for leftward motions) and the adjacent “S” (for rightward motions). For participants that sat on the right, they used the “K” (for leftward motions) and the adjacent “L” (for rightward motions). Responses were performed on the two keyboards placed in front of the participants (i.e., participants sitting on the left used the left keyboard, whereas participants sitting on the right used the right keyboard). While participants performed this task, they were also required to fixate the central dot on the computer screen. We did not explicitly instruct participants to turn their heads to the computer screen but often observed this to be the case as it is a more natural head position to fixate the central dot. To directly align their heads with the center of the screen, participants likely turned their heads by about 25 degrees. To ensure that participants maintained fixation, similar to Heed et al. (2010), there were a small number of catch trials (11 %), in which the central fixation dot would briefly flash (50 ms) and no other stimuli were presented. When this happened, the participant was required to press the “space” key if they sat on the left and the “enter” key if she/he sat on the right (“fixation control task”). Participants were instructed to prioritize accuracy over speed for their responses. As a point of note, any of the keys would end a trial regardless of the required task.

The other participant in the pair was required to perform the fixation control task and indicate the movement direction of the flashes (“visual motion discrimination task”). Again, depending on the seating position, either the “enter” or “space” key would be required for the fixation control task and either the “A” and “S” or the “K” and “L” keys would be required for indicating the moving direction of the visual stimuli.

**Figure 3:**
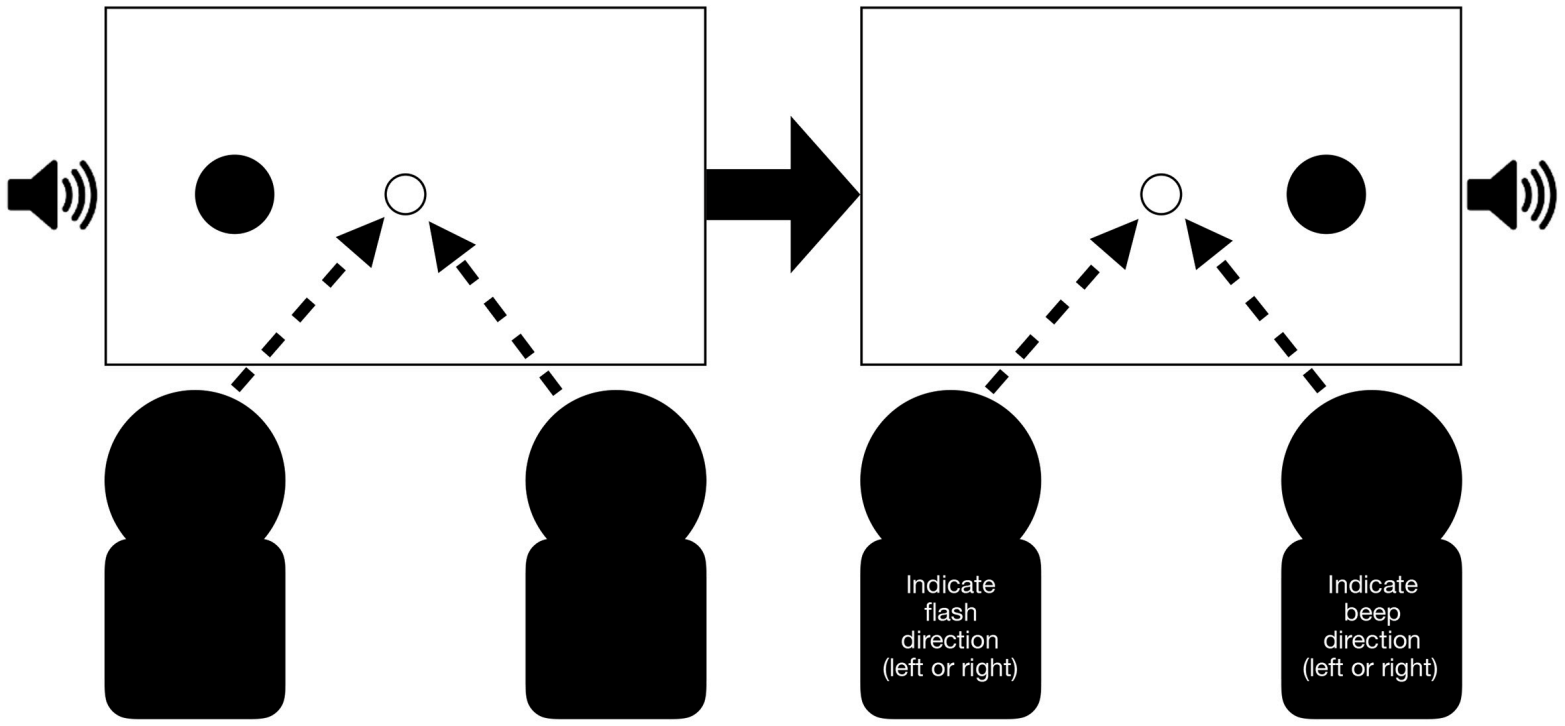
Example of a synchronous congruent trial for the joint condition: Participants receive a flash and beep presented on the left side followed by a flash and beep presented on the right side. In this example pair, the left participant is required to indicate the flash direction (left or right) while the participant sitting on the right is required to indicate the beep direction (left or right). Arrows indicate that participants were also required to fixate the center of the screen. A trial is completed after both participants pressed a key.

Participants performed their assigned tasks either alone or jointly (see Figure 3). When they were alone in the room, they sat in the same seat that they occupied when performing the task jointly. The seating positions of participants performing the different tasks were counterbalanced across pairs.

As a point of note, as it has been done in earlier studies (Heed et al., 2010; Wahn et al., 2017a), in the data results section we only considered the response data of the participant performing the auditory motion discrimination task, as the crossmodal effects were expected to occur in the auditory motion discrimination task (i.e., the visual stimuli were expected to influence the auditory motion discrimination but not vice versa).

Testing involved two sets of three blocks: visual discrimination performed alone, auditory motion discrimination performed alone (by the other participant), and visual and auditory discrimination tasks performed simultaneously by the two participants together. The order of the conditions in a set was randomly selected and then repeated. Each block had 56 trials, composed of 8 fixation control trials and 48 motion discrimination task trials. Each block was composed of an equal number of trials for each combination of the factor levels of Synchrony (synchronous, asynchronous) and Congruency (congruent, incongruent) trials (e.g., 12 synchronous congruent trials and 12 synchronous incongruent trials). After the last required response on a trial, the program automatically continued to the next trial following a 1,000 ms break.

At the beginning of each block, the block type was announced on the screen (“Joint Block,” “Individual Auditory Block,” or “Individual Visual Block”), and participants were asked to contact the experimenter. The experimenter would then make the necessary setup adjustment (e.g., ask one of the participants to wait outside of the experimental room). The experimenter waited outside of the experimental room throughout testing.

The experiment was programmed using Python 2.7.3. It took about 20 min to complete.

#### 2.3.2. Temporal Order Judgment Task

**Figure 4:**
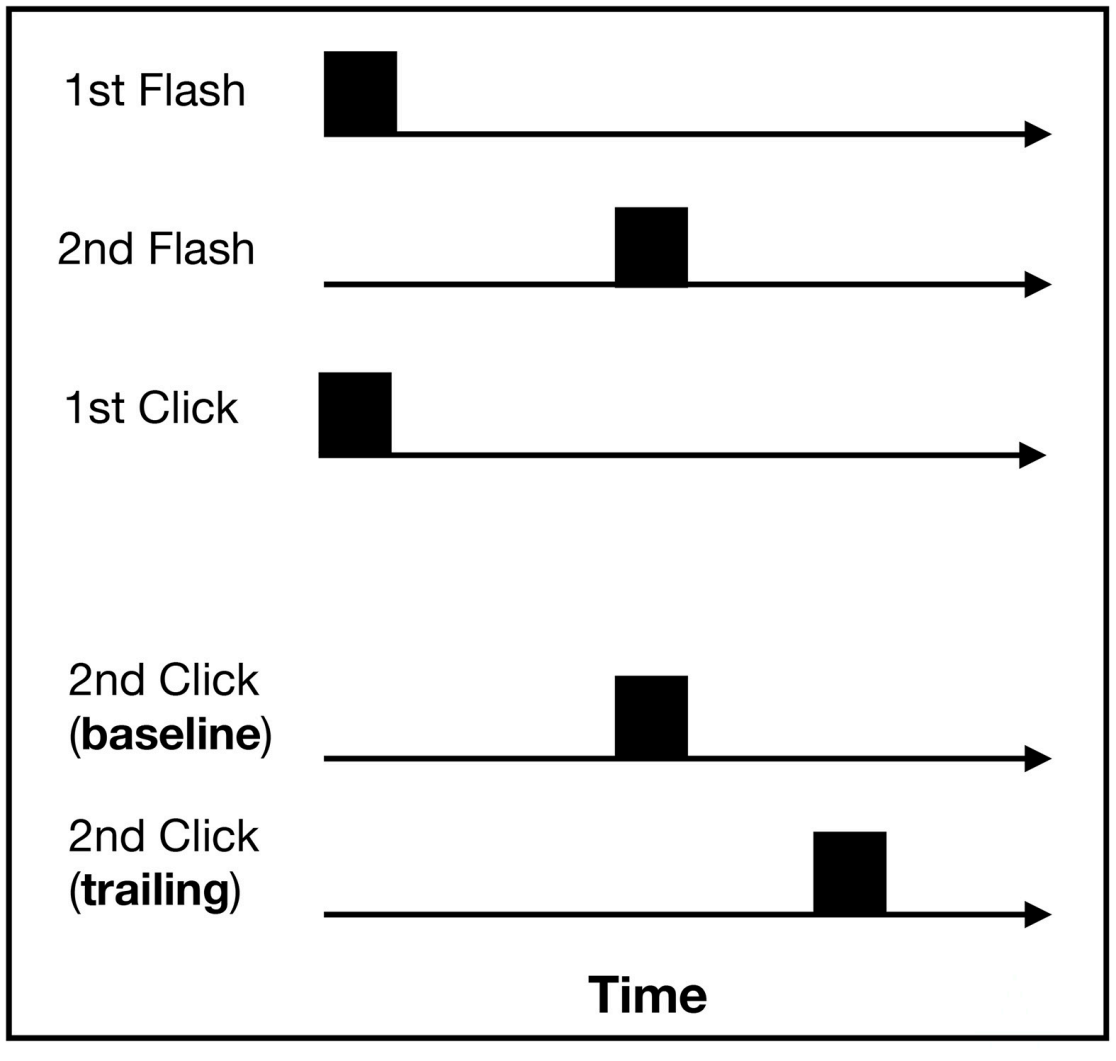
Stimuli combinations for the temporal order judgment task: two auditory and two visual stimuli were presented. The first visual and auditory stimulus was presented at the same time. The second auditory stimulus was either presented together with the second flash (baseline trial) or 100 ms after the second flash (trailing trial).

In the temporal order judgment task, two flashes (radius: 1.34 visual degrees, 5 ms) were presented in a rapid temporal sequence. The time between the flash presentations was randomly selected for each trial out of a set of four stimulus onset asynchronies (SOAs): 25, 50, 75, and 100 ms. One flash was presented below and one above the fixation dot (radius: 0.53 visual degrees) at a distance of 15 visual degrees. Whether the top or the bottom flash was presented first varied randomly between trials. Simultaneously with the first flash, a click sound (impulse tone, 5 ms) was presented as well. Depending on the type of trial, a second click was presented simultaneously with the second flash (baseline trial) or the second click trailed behind the second flash by 100 ms (trailing trial) (for an overview of all stimuli combinations, see Figure 4). One of the clicks was presented from the left loudspeaker and the other from the right loudspeaker. Baseline and trailing trials were selected randomly, as were which speaker delivered which sound. Given earlier findings by Morein-Zamir et al. (2003), we expected crossmodal effects to occur in the trailing trials as the trailing click should affect the temporal perception of the second flash, “pulling” the perception of the two flashes apart.

As a point of note, the stimuli presentations of the clicks (left and right) and flashes (top and bottom) were deliberately chosen to be orthogonal to avoid any spatial crossmodal influences between stimuli. Moreover, contrary to the stimuli presentations in the motion discrimination task above, the flashes remained on the screen after stimulus onset and only disappeared after the participants' responses. Also note that we deviated with regard to a few design choices from the original study by Morein-Zamir et al. (2003). The original study selected SOAs from a larger set with a wider range (12, 24, 36, 48, 72, 96, and 144 ms). We selected SOAs from a smaller range and set (25, 50, 75, and 100 ms) to reduce the overall number of trials. Moreover, the original study included several types of trailing trials (ranging from 75 to 600 ms). We only used one type of trailing trials, for which the crossmodal effect was the strongest in the original study (100 ms).

As for the motion discrimination task above, each participant in a pair was assigned to perform a task in one of the sensory modalities. In particular, one participant in the pair was required to indicate whether the upper or lower flash occurred first (visual temporal order judgment task), which was the same task as performed by participants in the original study (Morein-Zamir et al., 2003). If the participant was sitting on the left, she/he was required to use the “A” key to indicate that the top flash came first and the “Z” key to indicate that the bottom flash occurred first. If the participant was sitting on the right, she/he was required to use the “K” key to indicate that the top flash occurred first and the “M” key to indicate that the bottom flash occurred first. While performing the visual temporal order judgment task, the participant was also required to maintain fixation on the central fixation dot. As for the motion discrimination task above, during a small number of trials (3%), the participant was also required to perform the fixation control task. That is, the central fixation dot would briefly flash (50 ms) and no other stimuli were presented during such a trial. Also, for this task, we did not explicitly instruct participants to turn their heads toward the computer screen but often observed this to be the case as it is a more natural head position to fixate the central dot. For these fixation control task trials, depending on the seating position, the participant was required to press “space” (sitting of the left) or “enter” (sitting on the right).

The other participant in the pair was required to indicate which of the clicks occurred first (auditory temporal order judgment task). If the participant was sitting on the left, pressing the “A” key would be required to indicate that the click played on the left speaker occurred first and the adjacent “S” key when the click played on the right speaker occurred first. If the participant was sitting on the right, the “K” (for left) and “L” (for right) were required. While performing the auditory temporal order judgment task, the participant was also required to maintain fixation at the central dot and to also perform the fixation control task. As for the motion discrimination task above, all participants were instructed to prioritize accuracy over speed for their responses for all tasks.

As for the motion discrimination task above and in line with earlier studies investigating the effect of joint task performance on multisensory integration (Heed et al., 2010; Wahn et al., 2017a), we only considered the response data of participants performing the visual temporal order judgment task since crossmodal effects were only expected to occur in this task. Indeed, like Morein-Zamir et al. (2003), we did not collect trailing visual stimuli to assess the influence of visual signals on auditory temporal order judgments.

**Figure 5:**
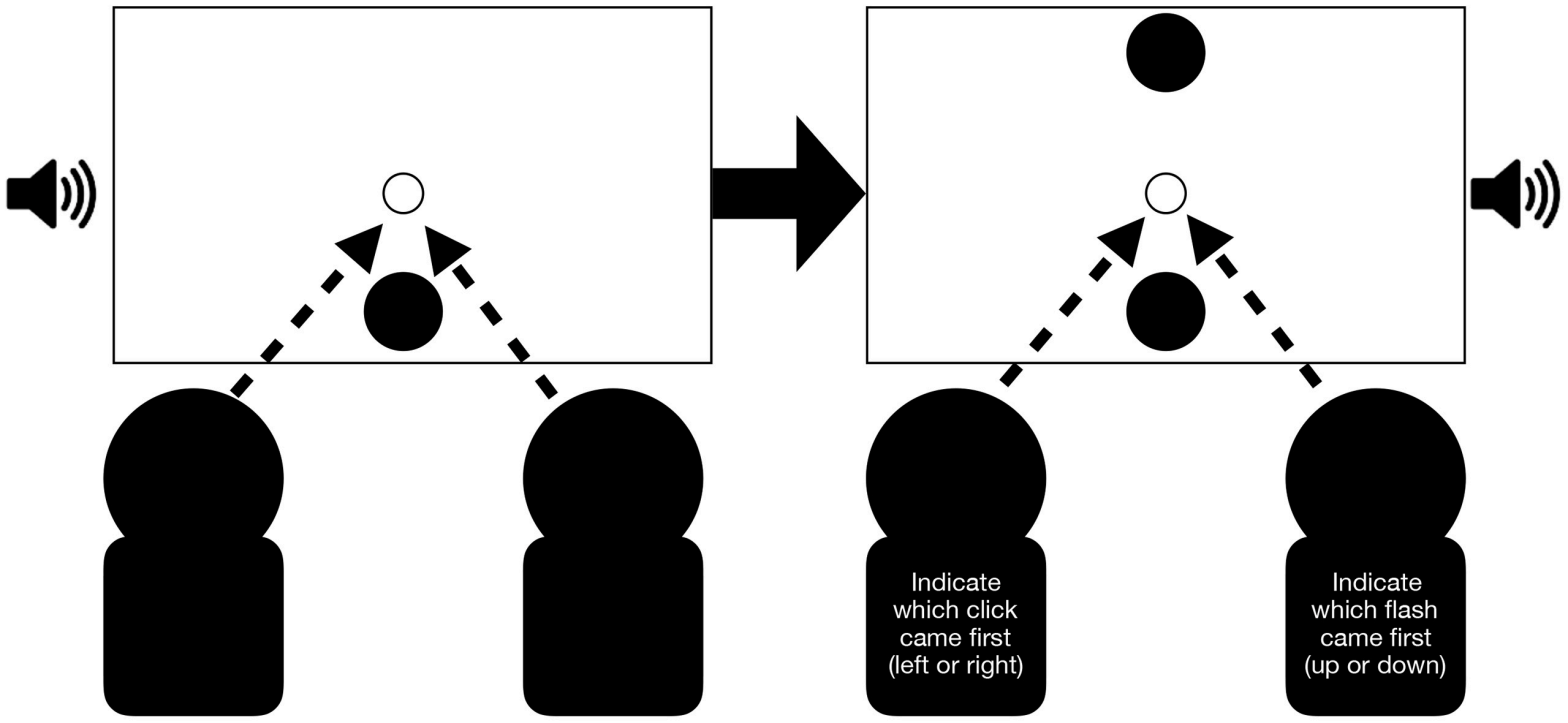
Example of baseline trial in the joint condition: Participants first receive a flash presented at the bottom and simultaneously a click sound on the left. Then, a second flash is presented (the first one remains on the computer screen) simultaneously with a second click on the right. In this example seating arrangement, the left participant is required to indicate which click came first and the right participants is required to indicate which flash came first. Arrows indicate that participants were also required to fixate the center of the screen. A trial is completed after both participants pressed a key.

As before, the experiment was divided into two sets of three blocks: one block for each participant to perform the visual or auditory temporal order judgment task while alone in the room and one block for the two tasks to be performed simultaneously while together in the room (see Figure 5). Each block contained 136 trials. Four of these were trials for the fixation control task. Half of the remaining trials were trailing trials, and the other half were baseline trials. After responses were made, the program automatically continued to the next trial after a 1,000 ms break. Again, the experimenter waited outside of the experimental room throughout testing, making the necessary setup adjustment between blocks.

The experiment took about 40 min to complete. It was programmed in Python 2.7.3.

### 2.4. Data Pre-processing

For our data analysis later on, in line with earlier studies and as noted in the task procedure, we only considered data of the participants performing the tasks in which crossmodal effects were expected to occur (i.e., the auditory motion discrimination task and visual temporal order judgment task).

To briefly confirm this expectation, at least for the motion task (as an analysis for the auditory temporal order judgment task is not feasible as noted above), we assessed the performance in the visual motion discrimination task for the synchronous individual condition and found a high accuracy performance regardless of the type of presentations (congruent: *M* = 0.92 vs. incongruent: *M* = 0.90). We also found that there was no significant difference between congruent and incongruent presentations [*t*_(11)_ = –1.16, *p* = 0.269], suggesting that there were no crossmodal effects present in the visual motion discrimination task. In the following, only data from the auditory motion discrimination and visual temporal order judgment task were considered.

To assess participants' general performance accuracy for the two tasks we primarily considered for the analysis (i.e., the auditory motion discrimination task and visual temporal order judgment task), we used baseline data from the conditions where no crossmodal effects were expected to occur. For the auditory motion discrimination task in particular, we used the data from the incongruent asynchronous presentations when a participant performed their task alone in the room. For the visual temporal order judgment task, we used the baseline trials with the longest SOA (100 ms) when a participant was alone in the room. For both these situations, and for the fixation control tasks, we set the inclusion criteria to a performance above 70%.

We aimed to match the sample size of our current study to the sample size of earlier studies investigating social manipulations in crossmodal tasks, which was 11 in the case of Heed et al. (2010) and 12 in the case of Wahn et al. (2017a). Moreover, we counterbalanced the seating position and task order across pairs such that we have an equal number of pairs for each combination of these factors. We also sought to have a sample of participants that were able to accurately perform the motion discrimination task, temporal order judgment task, and fixation control task. Our data collection ran until all these criteria were fulfilled for a sample size of 12 pairs (21 females and 3 males, *M* = 19.67 years, *SD* = 1.68 years). An additional 7 pairs (11 females, 3 males, *M* = 19.42 years, *SD* = 1.03 years) did not fulfill our inclusion criteria.

The fixation control task was performed at a high accuracy in the final sample both in the motion discrimination experiment (*M* = 97% correct, *SD* = 4.81%) and temporal order judgment experiment (*M* = 96% correct, *SD* = 3.08%).

## 3. Results

### 3.1. Auditory Motion Discrimination Task

Based on Soto-Faraco et al. (2002), we expected the factors Congruency and Synchrony to interact. The rationale is that the performance difference between incongruent and congruent presentations should be larger for the synchronous than the asynchronous presentations because it is during the synchronous presentations that the incongruent visual signals should distract participants from accurately judging the sound motion. For social factors to influence this crossmodal effect, we would expect a significant interaction between the factors Congruency, Synchrony, and Social Condition.

**Figure 6:**
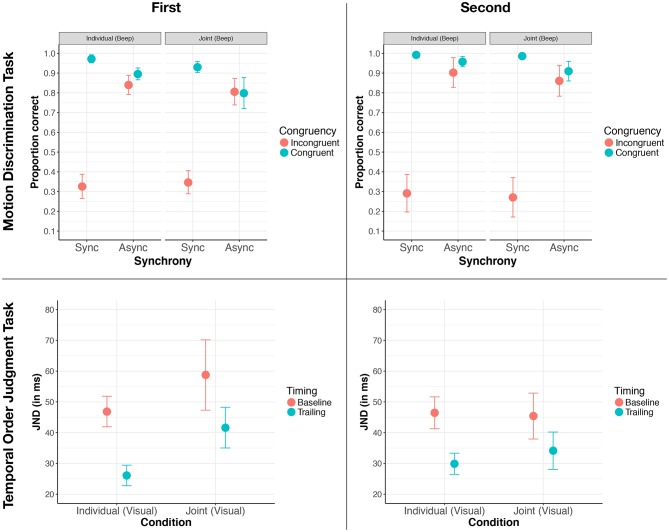
Results overview for the motion discrimination task (upper two panels) and the temporal order judgment task (lower two panels) when the respective task is performed first (left column) or second (right column). Error bars are standard error of the mean in all panels.

Figure 6 (upper panels) displays the response accuracy for all combinations of these factors, including the task order. On a descriptive level, we observed large differences between congruent and incongruent presentations for the synchronous trials, suggesting that the crossmodal effects that found in the original study Soto-Faraco et al. (2002) were replicated in the present study. With regard to our social manipulation, we observed that the difference between congruent and incongruent presentations for synchronous trials was not modulated by whether a task was performed alone or jointly. Lastly, we did not observe any order effects (i.e., that the pattern of results for performing the motion discrimination task first or second did not differ).

To confirm whether these observations were significant, we analyzed participants' performance using a four-factorial ANOVA with the response accuracy as the dependent variable. The three within-subject factors were Congruency (Incongruent and Congruent), Synchrony (Synchrony and Asynchrony), and Social Condition (Alone and Joint). The between-subject factor was the Task Order (First and Second). We found main effects for the factors Synchrony [*F*_(1, 10)_ = 41.60, *p* < 0.001, $\eta_{G}^{2}=$ 0.43] and Congruency [*F*_(1, 10)_ = 65.55, *p* < 0.001, $\eta_{G}^{2}=$ 0.63]. Replicating crossmodal effects from the original study (Soto-Faraco et al., 2002), we found an interaction effect between these two factors [*F*_(1, 10)_ = 184.27, *p* < 0.001, $\eta_{G}^{2}=$ 0.58]. Given that our ANOVA only involved factors with two levels, there were no follow-up tests required since the interaction effect already tested the pairwise comparison. To describe this interaction in more detail, the difference in performance accuracies between congruent and incongruent presentations was significantly larger for synchronous presentations than for asynchronous presentations. All other effects in the ANOVA were not significant (all *ps* > 0.129).

As the absence of a significant interaction effect between the factors Congruency, Synchrony, and Condition [*F*_(1, 10)_ = 0.11, *p* = 0.746] suggested that performing a task jointly did not affect audiovisual integration, we also computed a Bayes factor using the R package “BayesFactor” (Morey et al., 2015) for this effect to assess how much more likely the null hypothesis was relative to the alternative hypothesis. We found a Bayes factor of 0.30, meaning that our data was 1/0.30 or 3.33 times more likely under the null hypothesis than the alternative hypothesis.

To test for the possibility of whether an auditory stimulus moving toward where the participant was sitting or away from the participant may have interacted with our Social Condition factor, we repeated the above ANOVA with the additional factor Auditory Moving Direction (Away and Toward). Apart from the effects already found above (i.e., a significant main effects for the factors Congruency [*F*_(1, 10)_ = 65.55, *p* < 0.001, $\eta_{G}^{2}=$ 0.54] and Asynchrony [*F*_(1, 10)_ = 41.60, *p* < 0.001, $\eta_{G}^{2}=$ 0.34] and a significant interaction effect between these two effects [*F*_(1, 10)_ = 184.27, *p* < 0.001, $\eta_{G}^{2}=$ 0.49], no other effects in the ANOVA were significant (all *ps* > 0.068).

Apart from assessing response accuracy, we also repeated the same ANOVA above for the response times as the dependent variable. We found no significant effects for this ANOVA. In particular with regard to crossmodal effects, there was no significant interaction effect between the factors Congruency and Synchrony [*F*_(1, 10)_ = 1.18, *p* = 0.302], suggesting there were no crossmodal effects present from the perspective of the response times.

In sum, we replicated earlier crossmodal effects by Soto-Faraco et al. (2002), finding that for synchronous presentations visual stimuli affected auditory motion judgments more than for asynchronous presentations. We did not find that performing a task jointly modulated this effect. In fact, our calculated Bayes factor suggests that the null hypothesis that there is no effect is considerably more likely than the alternative hypothesis.

### 3.2. Visual Temporal Order Judgment Task

For analyzing the data of the visual temporal order judgment task, we followed the same analysis procedure as in the original study by Morein-Zamir et al. (2003). That is, using a logistic regression, we fitted psychometric curves to each of the participants' responses, separately for each condition. Based on these fits, we extracted for each participant the just noticeable difference (JND). These JNDs were used as a dependent variable for our further analyses.

To replicate the crossmodal effect found by Morein-Zamir et al. (2003), we expected a main effect for the factor Timing with the levels baseline and trailing. That is, we expected that participants were significantly better at judging which of the two flashes occurred first for trailing trials compared to baseline trials. For a social effect to occur, we expected an interaction effect between the factors Social Condition (Alone, Joint) and Timing (Baseline, Trailing). Plotting the averaged JNDs as a function of these factors, including Task Order (see Figure 6, lower panels), we observed large differences between baseline and trailing trials, suggesting that we replicated the earlier crossmodal effect. Yet, the size of this crossmodal effect (i.e., the difference in JND between baseline and trailing trials) did not appear to be modulated by whether the task was performed jointly or alone. However, there was the suggestion that the effect of the Social Condition was modulated by Task Order, with the JNDs somewhat raised for the joint condition compared to the individual condition when the temporal order judgment task was performed first.

To assess whether these observations were statistically significant, we performed a three factorial ANOVA with the JNDs as the dependent variable and the within-subject factors Social Condition (Alone, Joint) and Timing (Baseline, Trailing) and the between-subject factor Task Order. We found a main effect of Timing [*F*_(1, 10)_ = 39.05, *p* < 0.001, $\eta_{G}^{2}=$ 0.24]. All the other effects were not significant (all *ps* > 0.124). There were no interaction effects, including, most importantly, no significant Social Condition x Timing interaction effect, [*F*_(1, 10)_ = 1.06, *p* = 0.327]. For this interaction, we again computed a Bayes factor to assess how more likely the null hypothesis is compared to the alternative hypothesis given the present data. We observed a Bayes factor of 0.47, meaning that our data were 1/0.47 or 2.15 times more likely under the null hypothesis than the alternative hypothesis.

In sum, we replicated the crossmodal effect found by Morein-Zamir et al. (2003). As for the motion discrimination task above, we found that audiovisual integration was not affected by performing the task jointly rather than alone. In fact, the null hypothesis that there is no effect was more than two times more likely than the alternative hypothesis.

Similar to the auditory motion discrimination task, we also tested whether the click starting position (either starting on the participant's side or opposite side) interacted with our social manipulation. For this purpose, we repeated the ANOVA above with the additional factor Auditory Starting Position (Same and Opposite). We again found a significant main effect of Timing [*F*_(1, 10)_ = 67.02, *p* < 0.001, $\eta_{G}^{2}=$ 0.26]. Moreover, we found a significant main effect of Auditory Starting Position [*F*_(1, 10)_ = 7.70, *p* = 0.012, $\eta_{G}^{2}=$ 0.03] and a significant interaction effect between Timing and Auditory Starting Position [*F*_(1, 10)_ = 7.84, *p* = 0.019, $\eta_{G}^{2}=$ 0.03]. Yet, none of the effects involving the factor Social Condition (Alone and Joint) were significant (all *ps* > 0.155).

Given that we replicated earlier crossmodal effects in both tasks, we also correlated the sizes of these effects across tasks. For each task we averaged the data across the levels of all factors except for Timing in the temporal order judgment task and Congruency in the motion discrimination task. For the temporal order judgment task, we then computed the difference between the baseline and trailing condition. For the motion discrimination task, we computed the difference between the congruent and incongruent condition. Correlating these differences, we found a moderately sized correlation, which was not significant [*r* = –0.39, *t*_(10)_ = –1.35, *p* = 0.204]. For this correlation, we found a Bayes factor of 0.80, meaning that our data were 1/0.80 or 1.15 times more likely under the null hypothesis than the alternative hypothesis.

## 4. Discussion

In the present study, we replicated earlier crossmodal effects in a motion discrimination task (Soto-Faraco et al., 2002) and temporal order judgment task (Morein-Zamir et al., 2003); auditory judgments of motion were affected by visual input, and visual judgments of timing were affected by auditory input. In the case of the motion discrimination task, we found that participants' performances were significantly worse when incongruent rather than congruent visual information was presented. For the temporal order judgment task, participants were better at judging the order of the flashes if a click trailed the second flash. These findings demonstrate that these known crossmodal effects were robust, persisting despite the many design changes we made, the most profound ones being that we introduced social situations where two participants performed their respective tasks together.

On this last score, despite the introduction of joint task situations, and contrary to previous findings that a joint performance modulated crossmodal spatial congruency effects (Heed et al., 2010; Wahn et al., 2017a), we found that our robust crossmodal motion and timing effects were not modulated by joint task performance. These findings highlight the importance of investigating the effects of social factors across a range of crossmodal paradigms. In other words, as we had noted in the introduction, one cannot assume that because social factors impact crossmodal performance in one particular paradigm, social factors will affect crossmodal performance in all situations. Below, we speculate why the present tasks are resistant to social manipulations and how one might test our proposals in the future.

A major difference between the present task and crossmodal congruency tasks investigated earlier is that crossmodal effects in the present study were quantified in both tasks via perceptual judgments (i.e., response accuracies), while in the earlier studies effects were quantified (at least in part) with response times. Moreover, in the audiovisual crossmodal congruency task investigated earlier (Wahn et al., 2017a), the effect of task co-performance was also only present for the response times while response accuracies were unaffected. Given that response accuracies were also not affected by joint task performance in the present study for both tasks, one could also suggest that performing a task jointly specifically affected crossmodal effects quantified with response times while performance accuracies remain unaffected. An outstanding question for future investigation is whether those past response time crossmodal effects reflect multisensory integration at all or merely the speed at which one of the signals reaches the response threshold. The fact that no social effect has been observed in response accuracy suggests that there may not be any social effect on multisensory integration.

An alternative explanation for the divergent findings between the present data and previous work may rest with the difference in task demands between the present study and earlier studies. In particular, in earlier studies participants were required to localize static stimuli whereas in the present study participants were required to judge the movement direction of stimuli and their temporal order. Given this difference between tasks, one possibility could be that participants are only able to “off-load” stimuli to a co-actor if the task primarily involves static spatial stimuli (as in the earlier studies Heed et al., 2010; Wahn et al., 2017a) while this is not possible for moving stimuli or temporal stimuli. In other words, stimuli may be required to be spatial and static for co-actors to be able to “off-load” these stimuli to other co-actors. Possibly, the mechanism of task co-representation, which was suggested to have allowed participants to filter out distracting stimuli in earlier studies; (Heed et al., 2010; Wahn et al., 2017a) could be specific to static spatial stimuli. Future studies could test this proposal by investigating whether joint task performance also does not affect multisensory integration in other tasks requiring spatial processing of moving stimuli (e.g., in an audiovisual bounce-inducing effect; Sekuler, 1997; Grassi and Casco, 2009) or temporal processing (e.g., in the sound-induced flash illusion; Shams et al., 2002).

Related to the difference in task demands between the present study and those earlier, whether joint task performance affects multisensory integration or not may also depend on the strength of the integration of the investigated multisensory effect. In particular, for the motion discrimination task, the integrated moving stimuli may more strongly be integrated (and hence less susceptible to effects of joint task performance) as the received stimuli provide more cues (i.e., spatial *and* motion information) to be integrated. Similarly, for the temporal order judgment task, the mere fact of presenting two audiovisual stimuli may have resulted in a stronger integration of stimuli that is less susceptible to the effects of joint task performance. Future studies could test this proposal by investigating whether joint performance task performance also does not affect multisensory integration in other tasks that involve more richer audiovisual stimuli (than static spatial stimuli). Another difference to consider, which is specific to the temporal order judgment task in relation to the spatial congruency tasks in earlier studies (Heed et al., 2010; Wahn et al., 2017a), concerns the direction of the crossmodal effects (i.e., whether stimuli in the visual sensory modality influence processing stimuli in a different sensory modality or vice versa). That is, in the temporal order judgment task crossmodal effects were present for stimuli in the visual sensory modality (i.e., auditory information influenced visual processing) whereas in the earlier studies crossmodal effects were either present in the auditory or tactile sensory modalities due to an influence of visual stimuli. Hence, one could raise the possibility that joint task performance only affects multisensory integration in tasks, where the visual sensory modality is affecting processing in other sensory modalities but not vice versa. Yet, given that we did not find an effect of joint task performance for the audiovisual motion discrimination task—a task in which visual stimuli affect auditory processing—this proposal may only apply to static stimuli in crossmodal tasks.

In summary, we successfully replicated earlier crossmodal temporal and motion effects, in which participants were required to perform perceptual judgments. Yet, the present work fails to find evidence that joint task performance modulates these replicated crossmodal effects. Given that earlier studies found an effect of joint task performance for crossmodal spatial congruency tasks (Heed et al., 2010; Wahn et al., 2017a), we suggest that the effect of joint task performance on crossmodal tasks could potentially depend on how crossmodal effects are quantified (i.e., via responses times or accuracies) and task demands (i.e., whether tasks require processing stimuli in terms of location, motion, or timing).

## Data Availability Statement

The datasets collected for this study are available on request to the corresponding author.

## Ethics Statement

The studies involving human participants were reviewed and approved by University of British Columbia's ethics committee. The participants provided their written informed consent to participate in this study.

## Author Contributions

BW, JD, and AK: Study design, wrote the manuscript and revised the manuscript. BW: Programmed the experiments and analyzed the data.

### Conflict of Interest

The authors declare that the research was conducted in the absence of any commercial or financial relationships that could be construed as a potential conflict of interest.
